# Adjunctive Corticosteroids in Pediatric Meningitis: Current Evidence, Clinical Controversies, and Future Directions

**DOI:** 10.3390/ph19101563

**Published:** 2026-10-01

**Authors:** Giulia Zambelli, Marco Masetti, Sonia Diona, Lorenzo Bonacorsi, Irene Addati, Susanna Esposito

**Affiliations:** Pediatric Clinic, Department of Medicine and Surgery, University Hospital of Parma, 43126 Parma, Italy; giulia.zambelli@unipr.it (G.Z.); marco.masetti@unipr.it (M.M.); sonia.diona@unipr.it (S.D.); lorenzo.bonacorsi@unipr.it (L.B.); irene.addati@unipr.it (I.A.)

**Keywords:** bacterial meningitis, pediatric meningitis, corticosteroids, dexamethasone, adjunctive therapy, neurological sequelae, sensorineural hearing loss, mortality, neuroprotection

## Abstract

**Background:** Pediatric bacterial meningitis remains an important cause of death and long-term neurological disability despite advances in vaccination, antimicrobial therapy, and supportive care. Because host inflammation contributes substantially to secondary neuronal injury, adjunctive corticosteroids have been investigated as a strategy to reduce inflammation-mediated complications. This narrative review aims to critically evaluate the current evidence on corticosteroid therapy in pediatric meningitis, with particular emphasis on acute bacterial meningitis, major clinical and neurological outcomes, treatment timing, pathogen-specific effects, and the impact of changing post-vaccination epidemiology. **Methods:** A structured search of PubMed/MEDLINE, Scopus, and Web of Science was conducted for relevant publications available up to June 2026. Randomized controlled trials, observational studies, systematic reviews, meta-analyses, and international guidelines involving patients aged 0–18 years were considered. Evidence was synthesized narratively, focusing on mortality, hearing loss, neurological sequelae, long-term outcomes, safety, causative pathogen, and timing of corticosteroid administration. **Results:** Adjunctive dexamethasone has not demonstrated a consistent mortality benefit, while evidence concerning hospital stay, intensive care utilization, and healthcare costs remains limited. Its most reproducible benefit is a reduction in sensorineural hearing loss, particularly in *Haemophilus influenzae* type b (Hib) meningitis. Benefits in pneumococcal meningitis are less consistent, and evidence supporting routine use in meningococcal disease remains limited. Efficacy appears greatest when dexamethasone is administered before or with the first antibiotic dose and may be less pronounced in the post-vaccination era. In tuberculous meningitis, corticosteroids improve survival, whereas routine use is not supported in uncomplicated viral meningitis or cryptococcal meningitis. **Conclusions:** Adjunctive dexamethasone should be used selectively in pediatric meningitis, with its clearest benefit being hearing preservation in early-treated Hib meningitis. Treatment decisions should account for pathogen, timing, and epidemiological context.

## 1. Introduction

Meningitis comprises a heterogeneous group of infectious and non-infectious inflammatory disorders of the meninges. Acute bacterial meningitis is its most severe form and remains an important cause of mortality and long-term neurological disability, particularly in children [1].

Neurological injury results from both microbial invasion of the central nervous system and the subsequent host inflammatory response. After entering the subarachnoid space, pathogens proliferate in the cerebrospinal fluid (CSF) and trigger the release of pro-inflammatory cytokines and chemokines, including interleukin-1β, tumor necrosis factor-α, CXCL1, CXCL2, and CXCL5 [2,3,4,5,6,7,8,9]. These mediators promote neutrophil recruitment, oxidative and proteolytic injury, and increased blood–brain barrier permeability [2,3]. The resulting cerebral edema, increased intracranial pressure, vascular injury, impaired cerebral perfusion, and neuronal apoptosis contribute to complications such as sensorineural hearing loss, cognitive impairment, epilepsy, and motor deficits [6,7,8,9,10] (Figure 1).

Because inflammation contributes substantially to neurological injury, corticosteroids—particularly dexamethasone—have been investigated as adjuncts to antimicrobial treatment. Their aim is to attenuate the early inflammatory response, especially that intensified by antibiotic-induced bacterial lysis. However, clinical benefits in children are heterogeneous and appear to depend on the causative pathogen, timing of administration, disease severity, epidemiological setting, and healthcare context.

This narrative review critically evaluates the evidence for adjunctive corticosteroid therapy in pediatric meningitis, with particular emphasis on acute bacterial meningitis. It examines effects on mortality, sensorineural hearing loss, neurological sequelae, and long-term outcomes; evaluates the influence of treatment timing, causative pathogen, and vaccination-era epidemiology; and discusses current indications, controversies, emerging strategies, and research priorities. Corticosteroid use in tuberculous, viral, and fungal meningitis is also considered to clarify the settings in which treatment is supported, uncertain, or potentially inappropriate.

## 2. Methods

### 2.1. Review Design and Scope

This narrative review aimed to critically summarize the evidence concerning adjunctive corticosteroid therapy in pediatric meningitis, with particular emphasis on acute bacterial meningitis and its effects on mortality, sensorineural hearing loss, neurological sequelae, and other clinically relevant outcomes. The review also considered corticosteroid use in tuberculous, viral, and fungal meningitis to clarify the circumstances in which treatment may be beneficial, uncertain, or potentially harmful.

The review was designed as a clinically oriented narrative synthesis rather than a systematic review or meta-analysis. Accordingly, no protocol was registered, no formal risk-of-bias assessment was undertaken, and reporting was not structured according to the Preferred Reporting Items for Systematic Reviews and Meta-Analyses (PRISMA) statement. Nevertheless, the literature search, eligibility criteria, study-selection process, and evidence-extraction procedures are reported in sufficient detail to improve transparency and reproducibility.

### 2.2. Information Sources and Search Dates

A structured literature search was conducted in PubMed/MEDLINE, Scopus, and Web of Science. The final database search was performed on 30 April 2026 and covered publications available from database inception through that date. The electronic search was supplemented by manual examination of the reference lists of relevant reviews, systematic reviews and meta-analyses, international clinical practice guidelines, and landmark studies. Citation searching was also used to identify pertinent publications not retrieved through the electronic database searches.

The search was restricted to articles published in English.

### 2.3. Database Search Strategies

The search strategy combined controlled vocabulary, where available, with free-text terms relating to meningitis, the pediatric population, and corticosteroid therapy. Searches were adapted to the indexing requirements and syntax of each database. Outcome terms were not made mandatory because their inclusion could have excluded relevant studies in which mortality, hearing impairment, or neurological outcomes were reported only within the full text.

The complete search strategies were as follows: PubMed/MEDLINE ((“Meningitis”[Mesh] OR meningitis[Title/Abstract] OR “bacterial meningitis”[Title/Abstract] OR “tuberculous meningitis”[Title/Abstract] OR “viral meningitis”[Title/Abstract] OR “fungal meningitis”[Title/Abstract]) AND (“Adrenal Cortex Hormones”[Mesh] OR corticosteroid*[Title/Abstract] OR glucocorticoid*[Title/Abstract] OR dexamethasone[Title/Abstract] OR methylprednisolone[Title/Abstract] OR prednisolone[Title/Abstract] OR hydrocortisone[Title/Abstract]) AND (“Child”[Mesh] OR “Infant”[Mesh] OR “Adolescent”[Mesh] OR pediatric*[Title/Abstract] OR paediatric*[Title/Abstract] OR child*[Title/Abstract] OR infant*[Title/Abstract] OR adolescen*[Title/Abstract] OR neonat*[Title/Abstract])); Scopus TITLE-ABS-KEY (meningitis OR “bacterial meningitis” OR “tuberculous meningitis” OR “viral meningitis” OR “fungal meningitis”) AND TITLE-ABS-KEY (corticosteroid* OR glucocorticoid* OR dexamethasone OR methylprednisolone OR prednisolone OR hydrocortisone) AND TITLE-ABS-KEY (pediatric* OR paediatric* OR child* OR infant* OR adolescen* OR neonat*); Web of Science Core Collection TS = (meningitis OR “bacterial meningitis” OR “tuberculous meningitis” OR “viral meningitis” OR “fungal meningitis”) AND TS = (corticosteroid* OR glucocorticoid* OR dexamethasone OR methylprednisolone OR prednisolone OR hydrocortisone) AND TS = (pediatric* OR paediatric* OR child* OR infant* OR adolescen* OR neonat*).

The searches were conducted without filters for study design.

### 2.4. Eligibility Criteria

Publications were considered eligible when they examined corticosteroid therapy in infants, children, or adolescents aged 0–18 years with bacterial, tuberculous, viral, or fungal meningitis. Studies enrolling mixed pediatric and adult populations were considered only when pediatric findings were reported separately or when the pediatric population was directly relevant to the clinical question.

Eligible publication types comprised randomized controlled trials, prospective and retrospective cohort studies, case–control studies, systematic reviews, meta-analyses, and international or national clinical practice guidelines. Studies were required to report at least one outcome relevant to corticosteroid therapy, including mortality, sensorineural hearing loss, other neurological sequelae, neurodevelopmental or functional outcomes, length of hospitalization, intensive care utilization, inflammatory markers, treatment-related adverse events, or other measures of treatment effectiveness or safety.

Publications were excluded when they: exclusively examined adults and did not report pediatric findings separately; did not evaluate corticosteroid treatment or provide information relevant to its clinical use; did not report an outcome relevant to the objectives of the review; addressed meningitis only as part of a broader infection category without providing meningitis-specific findings; were case reports, small case series, editorials, letters without original or substantive evidence, conference abstracts without an accessible full report, or non-clinical studies that did not contribute directly to the pathophysiological rationale; or represented duplicate or superseded reports of the same study population, in which case the most complete or recent publication was retained.

Experimental and mechanistic studies were used selectively to explain the biological rationale for corticosteroid therapy but were not counted among the included clinical studies.

### 2.5. Literature Selection

Records retrieved from the three databases were combined and duplicates were removed using manual comparison of titles, authors, publication years, and digital object identifiers. Titles and abstracts were then assessed for relevance to the scope of the review. Potentially relevant articles underwent full-text assessment. Because this was a narrative review, study selection was based on relevance to the predefined clinical questions, methodological importance, and contribution to the interpretation of the evidence rather than on the exhaustive-selection framework required for a systematic review.

The database searches and supplementary reference-list screening identified the publications considered for this narrative review. Because study identification and selection were undertaken for a narrative rather than a systematic review, a formal screening log recording the number of records retrieved, duplicates removed, and articles excluded at each stage was not maintained. Consequently, retrospective numerical reconstruction of a PRISMA-style selection process was considered potentially inaccurate and was not undertaken.

### 2.6. Classification of the Included Evidence

The evidence base included randomized controlled trials, prospective and retrospective observational studies, systematic reviews and meta-analyses, and international or national clinical practice guidelines. These sources were selected according to their relevance to the predefined clinical questions, methodological importance, and contribution to the interpretation of corticosteroid use in pediatric meningitis.

### 2.7. Data Extraction

For each eligible primary study, information was extracted on study design, study setting, sample size, participant age, meningitis etiology, diagnostic criteria, corticosteroid agent, dosage, route and duration of administration, comparator, and timing of corticosteroid administration in relation to antimicrobial therapy. Extracted outcomes included mortality, sensorineural hearing loss, other neurological sequelae, neurodevelopmental and functional outcomes, length of hospitalization, intensive care unit admission or utilization, and treatment-related adverse events.

For systematic reviews and meta-analyses, the population, number and types of included studies, principal comparisons, outcomes, and main conclusions were recorded. For clinical practice guidelines, the issuing organization, publication year, target population, principal recommendations, and stated strength or certainty of evidence were documented.

Data were extracted by two reviewers independently (G.Z. and M.M.). Any uncertainties were resolved through discussion and consensus between reviewers. Because the purpose was narrative synthesis, no standardized risk-of-bias instrument or certainty-of-evidence framework was applied. Methodological limitations reported by the original studies and evidence syntheses were nevertheless considered when interpreting their findings.

### 2.8. Evidence Organization and Narrative Synthesis

The evidence was organized into predefined thematic domains: (i) the pathophysiological rationale for corticosteroid use; (ii) corticosteroid agents, regimens, and timing of administration; (iii) efficacy in pediatric bacterial meningitis; (iv) pathogen-specific effects; (v) corticosteroid use in non-bacterial meningitis; (vi) safety and adverse effects; (vii) short- and long-term clinical outcomes; and (viii) unresolved controversies and research priorities.

A quantitative meta-analysis was not undertaken because of substantial clinical and methodological heterogeneity in the available literature, including differences in patient age, causative pathogens, vaccination-era epidemiology, disease severity, corticosteroid regimens, treatment timing, healthcare settings, comparator groups, outcome definitions, and follow-up duration. Findings were therefore synthesized narratively, with particular attention to the consistency of results across study designs, the distinction between primary and secondary evidence, pathogen-specific treatment effects, timing of corticosteroid administration, and the applicability of historical findings to contemporary pediatric practice.

This approach was intended to provide a transparent, clinically oriented interpretation of the evidence while recognizing that the review was not designed to establish pooled effect estimates or provide the level of methodological completeness expected of a systematic review.

A study-level summary table was not prepared because the purpose of this narrative review was to integrate several distinct and partly overlapping evidence sources, including primary randomized and observational studies, systematic reviews and meta-analyses, and clinical practice guidelines, rather than to present an exhaustive systematic-review study set. Direct tabulation of these sources could imply equivalence between different levels of evidence and could result in double counting of primary populations represented within evidence syntheses. Transparency was instead supported by reporting the search and selection methods, specifying the information extracted from each source type, presenting quantitative results and methodological limitations for the principal studies and evidence syntheses in the text, and organizing the findings in clinically focused tables according to corticosteroid regimen, causative pathogen, outcomes, strength of evidence, safety, and practical implications.

## 3. Meningitis in Children

### 3.1. Epidemiology

Meningitis remains a major global health concern and continues to cause substantial morbidity, mortality, and long-term disability, particularly among children. Recent estimates indicate that approximately 2.5 million cases of meningitis occur worldwide each year, including nearly 1.6 million cases of bacterial meningitis and approximately 240,000 associated deaths [11,12].

The global burden of meningitis is unevenly distributed. The highest incidence is observed in low- and middle-income countries, particularly within the African “meningitis belt,” where recurrent outbreaks of meningococcal disease continue to occur [11,13]. By contrast, the incidence of bacterial meningitis has declined substantially in many high-income countries following the widespread introduction of conjugate vaccines targeting Hib, *Streptococcus pneumoniae*, and *Neisseria meningitidis* [14,15,16,17]. These vaccination programs have not only reduced overall disease incidence but have also altered the relative distribution of causative pathogens, with important implications for the contemporary role of adjunctive therapies. Conjugate vaccination has produced a particularly marked reduction in Hib disease. In the United States, the annual incidence of invasive Hib disease among children younger than 5 years has declined by approximately 99% since routine childhood vaccination was introduced. In 2023 supplemental surveillance covering approximately 5.5 million children younger than 5 years, the incidence of invasive Hib disease was 0.24 per 100,000; Hib accounted for 13 of 171 serotyped or reported invasive *H. influenzae* cases. This is an incidence estimate for all invasive Hib disease, not specifically Hib meningitis, but it illustrates the rarity of the organism in a highly vaccinated population. Most reported Hib cases with available vaccination information occurred in children who were unvaccinated or too young to have completed vaccination [18,19].

The post-vaccine etiological distribution differs substantially across regions. In the WHO-coordinated surveillance network, which included 58 countries and more than 137,000 suspected meningitis cases reported during 2014–2019, one of the three principal vaccine-preventable pathogens was identified in 3576 probable bacterial meningitis cases. Of these, *S. pneumoniae* accounted for 60.9%, *N. meningitidis* for 21.4%, and *H. influenzae* for 17.7%. Only 49.1% of serotyped *H. influenzae* isolates were type b. Pneumococcus therefore remained the most frequently detected of these three pathogens, although the proportion caused by pneumococcal vaccine serotypes declined as pneumococcal conjugate vaccine implementation increased. These data are derived largely from sentinel surveillance and should not be interpreted as a universal etiological distribution, but they demonstrate why historical Hib-dominated trials may not reflect current case mixtures [20].

Age is a major determinant of meningitis risk. Infants, particularly those younger than two months, experience the highest incidence because of immunological immaturity and increased susceptibility to invasive bacterial infection [21]. Although routine immunization has markedly reduced the burden of vaccine-preventable meningitis in older infants and children, bacterial meningitis continues to affect vulnerable populations, including very young infants, immunocompromised patients, incompletely vaccinated children, and those with limited access to timely healthcare.

Despite major advances in vaccination, antimicrobial therapy, and supportive care, bacterial meningitis remains associated with substantial mortality and neurological morbidity. Case-fatality rates may exceed 20–30% in some settings, and a considerable proportion of survivors develop permanent sequelae such as sensorineural hearing loss, epilepsy, cognitive impairment, and motor deficits [2,22,23,24,25]. Consequently, the burden of pediatric bacterial meningitis is determined not only by acute mortality but also by persistent neurological and neurodevelopmental disability. Prevention of inflammation-mediated neurological injury therefore remains an important therapeutic objective.

### 3.2. Etiology and Prognostic Factors

The etiology of meningitis varies according to age, immune status, vaccination history, geographical setting, and clinical context. Infectious causes predominate and include bacterial, viral, fungal, and parasitic pathogens, whereas non-infectious meningitis may occur in association with autoimmune diseases, malignancies, or drug-induced inflammatory reactions [1,26].

Among infectious etiologies, bacterial meningitis is associated with the greatest risk of rapid clinical deterioration, death, and permanent neurological sequelae. The distribution of causative organisms is strongly age-dependent. In neonates and young infants, Group B *Streptococcus* (*Streptococcus agalactiae*), *Escherichia coli*, and *Listeria monocytogenes* are among the principal bacterial pathogens, reflecting perinatal exposure and immature host defenses [27,28]. Although intrapartum antibiotic prophylaxis has substantially reduced early-onset Group B streptococcal disease, its effect on late-onset infection has been limited [29,30].

Beyond the neonatal period, *Streptococcus pneumoniae* and *Neisseria meningitidis* are the predominant causes of bacterial meningitis [23,31,32], whereas *Haemophilus influenzae* type b (Hib) remains important in settings where vaccination coverage is incomplete [2]. In immunocompromised children, *Listeria monocytogenes*, Gram-negative bacilli, and less common opportunistic pathogens account for a greater proportion of cases [1,33] (Table 1).

Viral meningitis is more common overall and generally follows a milder, self-limiting course. Nevertheless, bacterial meningitis remains responsible for most severe neurological complications and is therefore the principal focus of adjunctive corticosteroid therapy [1,34,35].

Most cases of bacterial meningitis arise following hematogenous dissemination of microorganisms and subsequent entry into the central nervous system. Direct extension from contiguous infectious foci, including sinusitis, mastoiditis, otitis, cranial trauma, or neurosurgical defects, may also occur [2,4].

Clinical manifestations vary considerably with age. Older children may present with fever, headache, neck stiffness, photophobia, vomiting, and altered mental status. In neonates, infants, and young children, manifestations are frequently nonspecific and may include temperature instability, irritability, poor feeding, lethargy, respiratory abnormalities, a bulging fontanelle, or seizures. This nonspecific presentation may delay recognition and treatment [1].

Early identification of children at increased risk of an adverse outcome is essential. Clinical features associated with severe disease include impaired consciousness, focal neurological deficits, recurrent or prolonged seizures, cranial nerve abnormalities, hemodynamic instability, respiratory compromise, and signs of increased intracranial pressure [36,37]. In pediatric cohorts, age younger than 12 months, pneumococcal etiology, delayed presentation, coma, and seizures have been associated with an increased risk of death, neurological complications, and long-term sequelae [2,23,38].

Laboratory findings may provide additional prognostic information. A low cerebrospinal fluid (CSF) leukocyte count despite severe clinical disease, particularly a count below 1000 cells/μL, hypoglycorrhachia, markedly elevated CSF protein concentrations, and positive blood cultures have been associated with poorer outcomes [1,39]. CSF lactate and serum procalcitonin may support differentiation between bacterial and viral meningitis when interpreted together with clinical findings and conventional CSF parameters [37,40]. No individual biomarker should, however, be used in isolation to confirm or exclude bacterial meningitis.

Etiology, disease severity, and prognosis are closely related to the potential effectiveness of adjunctive corticosteroids. The most consistent evidence of benefit has been reported in Hib meningitis, particularly for prevention of sensorineural hearing loss, whereas effects in pneumococcal and meningococcal disease are less uniform. These pathogen-specific differences reinforce the need to begin treatment promptly and subsequently reassess adjunctive therapy once microbiological results become available.

### 3.3. Initial Management

Acute bacterial meningitis is a medical emergency requiring immediate hospital admission or urgent transfer to a facility capable of providing lumbar puncture, parenteral antimicrobial therapy, cardiorespiratory monitoring, and management of severe neurological or systemic complications. Initial management should proceed concurrently rather than as a rigid sequence: physiological stabilization, collection of diagnostic specimens, and initiation of empirical treatment should occur with minimal delay (Table 2).

#### 3.3.1. Initial Assessment and Stabilization

The initial assessment should prioritize airway, breathing, circulation, neurological status, and recognition of sepsis or shock. Intravenous or intraosseous access should be established promptly. Oxygenation, blood pressure, heart rate, respiratory status, temperature, capillary glucose, urine output, and level of consciousness should be monitored. Hypoglycemia, hypoxemia, shock, electrolyte disturbances, and clinically apparent seizures require immediate treatment. Children with respiratory failure, persistent shock, recurrent seizures, rapidly deteriorating consciousness, or signs of intracranial hypertension should receive early pediatric intensive care assessment [1,12,36,39].

Droplet precautions should be instituted when meningococcal disease is suspected and maintained according to local infection-control guidance. Public health authorities should be notified promptly when meningococcal or other notifiable disease is suspected, allowing timely identification and chemoprophylaxis of eligible close contacts.

#### 3.3.2. Diagnostic Investigations

Blood cultures should be obtained as soon as possible, preferably before antimicrobial administration, together with a complete blood count, serum glucose, inflammatory markers, electrolytes, renal and hepatic function tests, and coagulation studies when clinically indicated. Obtaining diagnostic specimens must not delay empirical antimicrobial treatment.

Lumbar puncture should be performed as soon as clinically feasible, preferably before antibiotics, provided that the child is sufficiently stable and has no contraindication. Initial CSF evaluation should include opening pressure when practicable, macroscopic appearance, leukocyte count and differential, red blood cell count, protein, glucose with a paired blood glucose measurement, Gram staining, bacterial culture with antimicrobial susceptibility testing, and pathogen-specific molecular testing when available. CSF lactate may provide additional diagnostic information but should be interpreted alongside the overall clinical and laboratory findings [12,37,39,40].

Neuroimaging should not be performed routinely before lumbar puncture. Lumbar puncture should be deferred, and cranial imaging considered, in children with marked impairment of consciousness, focal neurological signs, cranial nerve deficits, papilledema, evidence of an intracranial mass or obstructive hydrocephalus, or another clinical concern for cerebral herniation. Lumbar puncture should also be deferred temporarily in the presence of cardiorespiratory instability, an uncontrolled bleeding disorder, or infection at the puncture site. When lumbar puncture is deferred or imaging is required, blood cultures should be obtained and antimicrobial treatment initiated immediately; treatment must not await imaging or CSF examination [1,12,36,39].

#### 3.3.3. Empirical Antimicrobial Therapy

Empirical intravenous antimicrobial therapy should be initiated as early as possible, ideally within 1 h of hospital assessment when bacterial meningitis is suspected. Diagnostic uncertainty, neuroimaging, transfer, or inability to perform lumbar puncture should not delay treatment [12,36,39].

Beyond the early-infant period, empirical therapy generally consists of a third-generation cephalosporin, such as ceftriaxone or cefotaxime. Vancomycin should be added where the prevalence of penicillin- or third-generation cephalosporin-resistant *S. pneumoniae* is sufficiently high or when resistance is otherwise suspected [2,41,42]. Local antimicrobial-resistance patterns and institutional guidance should inform the regimen.

Neonates and young infants require age-specific treatment that provides coverage for Group B *Streptococcus*, Gram-negative bacilli, and *L. monocytogenes*. Such regimens commonly include ampicillin combined with an appropriate cephalosporin, usually cefotaxime in neonates, or another agent selected according to neonatal treatment protocols and local susceptibility patterns [42,43,44]. Ceftriaxone requires particular caution in neonates because of bilirubin displacement and incompatibility with calcium-containing intravenous solutions.

Additional antimicrobial coverage may be required in immunocompromised children, patients with recent neurosurgery or penetrating cranial trauma, children with CSF shunts, and those with suspected hospital-acquired infection. Empirical antiviral therapy should be considered when herpes simplex encephalitis or meningoencephalitis cannot be excluded.

Once the causative organism and antimicrobial susceptibility profile are available, treatment should be narrowed to the most appropriate pathogen-directed regimen. Treatment duration should be individualized according to the pathogen, clinical response, complications, and adequacy of source control.

#### 3.3.4. Adjunctive Corticosteroid Therapy

When adjunctive dexamethasone is indicated, it should be administered immediately before or concomitantly with the first dose of antibiotics. The benefit is expected to be greatest when corticosteroid exposure precedes or coincides with antibiotic-induced bacterial lysis and the associated inflammatory burst. Antimicrobial administration should never be delayed to permit corticosteroid treatment [1,12,36,39].

Continuation of dexamethasone should be reassessed after microbiological identification. The strongest evidence supports continued treatment in Hib meningitis and, to a lesser and less consistent extent, pneumococcal meningitis. Routine continuation is generally not supported when another organism is identified, particularly when the potential benefit is absent or uncertain. Specific corticosteroid regimens and pathogen-dependent considerations are discussed in Section 4.

#### 3.3.5. Supportive Care and Management of Complications

Supportive management is an essential component of treatment. Isotonic fluids should be used when intravenous maintenance therapy is required, and routine fluid restriction is not recommended [43,44]. Fluid administration should instead be individualized according to perfusion, hydration, serum sodium, urine output, and the presence of cerebral edema or inappropriate antidiuretic hormone secretion.

Seizures should be treated promptly with age-appropriate antiseizure therapy [43,44]. Continuous or repeated electroencephalographic assessment may be appropriate in children with persistent altered consciousness or suspected non-convulsive seizures. Signs of increased intracranial pressure require urgent critical care and neurological assessment. Head elevation, adequate oxygenation and ventilation, avoidance of hypotension, and correction of metabolic disturbances form the basis of initial management [43,44]. Hypertonic saline or mannitol may be used as a temporary measure when acute intracranial hypertension is suspected, while definitive evaluation and treatment proceed. Glycerol should not be used routinely as adjunctive treatment.

Frequent reassessment is required to detect neurological deterioration, shock, inappropriate antidiuretic hormone secretion, electrolyte abnormalities, hearing impairment, and other systemic or neurological complications [43,44]. Before discharge, children should undergo neurological assessment and have audiological evaluation arranged, preferably before discharge or within 4 weeks. Children with hearing loss, motor impairment, cognitive difficulties, seizures, or other sequelae require early multidisciplinary follow-up and rehabilitation [43,44].

## 4. Adjunctive Corticosteroid Therapy in Pediatric Meningitis

### 4.1. Biological Rationale and Mechanisms of Action

Adjunctive corticosteroid therapy has been investigated primarily as a means of attenuating the excessive host inflammatory response responsible for a substantial proportion of neurological injury in meningitis. In bacterial meningitis, invasion of the subarachnoid space activates resident immune cells and induces the release of pro-inflammatory cytokines and chemokines. This response promotes leukocyte recruitment, disrupts blood–brain barrier integrity, and contributes to cerebral edema, increased intracranial pressure, vascular injury, impaired cerebral perfusion, and secondary neuronal damage [2,3,7,8].

The inflammatory response may be further intensified after antimicrobial treatment is initiated. Antibiotic-induced bacterial lysis releases cell-wall components and other pathogen-associated molecular patterns into the cerebrospinal fluid, amplifying cytokine production and downstream tissue injury [1,3,45]. Consequently, effective antimicrobial therapy may eradicate the infecting organism without completely preventing inflammation-mediated neurological complications.

Corticosteroids are therefore used as neuroprotective adjuncts rather than as agents with direct antimicrobial activity. Their principal effects include suppression of pro-inflammatory gene transcription, reduction in cytokine and chemokine production, inhibition of leukocyte migration into the cerebrospinal fluid, and attenuation of blood–brain barrier disruption [1,3]. These actions may reduce cerebral edema and secondary neuronal and cochlear injury, providing the biological rationale for administering corticosteroids before or with the first dose of antibiotics.

### 4.2. Corticosteroid Selection, Dosage, and Timing

Dexamethasone is the most extensively studied corticosteroid in acute bacterial meningitis and remains the preferred agent in major international guidelines [12,36]. Its use is supported by its potent anti-inflammatory activity, relatively long biological half-life, limited mineralocorticoid activity, and ability to penetrate inflamed meningeal tissues [39,45].

In children, a commonly recommended regimen is intravenous dexamethasone at a dose of 0.15 mg/kg every 6 h for 2–4 days [1,36,39]. Dexamethasone should be administered immediately before or concomitantly with the first dose of antibiotics. Antimicrobial therapy should never be delayed to permit corticosteroid administration.

The timing of treatment is a critical determinant of potential efficacy. Because the inflammatory cascade is rapidly amplified by antibiotic-induced bacterial lysis, corticosteroid administration after antimicrobial therapy has already begun is generally associated with reduced or absent benefit [39,45]. This timing dependency supports the interpretation of dexamethasone as an intervention directed against the early inflammatory burst rather than against an established phase of secondary neurological injury.

When dexamethasone is unavailable or cannot be administered, hydrocortisone or methylprednisolone may be considered at an equivalent glucocorticoid dose, in accordance with current WHO guidance [12,46,47,48]. Evidence supporting these alternatives in community-acquired pediatric bacterial meningitis is substantially more limited than that supporting dexamethasone. Prednisolone and hydrocortisone remain relevant in tuberculous meningitis, in which longer treatment courses and gradual tapering are generally required [48]. Fludrocortisone has no established anti-inflammatory role in pediatric meningitis, although its mineralocorticoid activity may be considered separately in selected patients with cerebral salt wasting.

The mechanisms, principal indications, limitations, and clinical considerations associated with the corticosteroids evaluated in meningitis are summarized in Table 3.

### 4.3. Pathogen-Specific Evidence in Acute Bacterial Meningitis

The clinical benefits of adjunctive corticosteroids are not uniform across bacterial pathogens. The strongest pediatric evidence for adjunctive dexamethasone concerns prevention of hearing loss in Hib meningitis. In the 2015 Cochrane review, corticosteroids reduced severe hearing loss in children with Hib meningitis (relative risk [RR] 0.34, 95% confidence interval [CI] 0.20–0.59), corresponding to hearing loss rates of approximately 4% vs. 12% [45]. This pathogen-specific result should nevertheless be interpreted cautiously. Most contributing trials were conducted during the 1980s and 1990s, before widespread Hib conjugate vaccination, and the numbers of children included in individual trials or pathogen-specific analyses were often limited. The studies also differed in dexamethasone dose, treatment duration, administration before or after the first antibiotic dose, antimicrobial regimens, definitions of hearing impairment, audiological methods, and follow-up duration.

Methodological quality was variable: of the 25 trials included in the Cochrane review, only four were classified as high quality, whereas 14 were of medium quality and seven were of low quality. Importantly, the reduction in severe hearing loss was not statistically evident when the analysis was restricted to high-quality studies [45]. The pooled Hib estimate may therefore be influenced by small-study effects, clinical heterogeneity, and older standards of antimicrobial and supportive care. These limitations do not negate the observed hearing-protection signal, but they reduce certainty regarding its magnitude and its direct applicability to contemporary vaccinated populations.

Evidence in pneumococcal meningitis requires separation of the all-age evidence from the pediatric evidence. In the 2015 Cochrane review, the pooled subgroup of participants with S. pneumoniae meningitis had lower mortality with corticosteroid treatment (29.9% vs. 36.0%; RR 0.84, 95% CI 0.72–0.98) [45]. However, this organism-specific analysis combined adults and children, and the review did not establish that the mortality effect was independently present in children. Furthermore, the analysis was based on etiological subgroups within trials rather than trials designed or powered specifically for pediatric pneumococcal meningitis. The pediatric meta-analysis by Tian et al., which included 29 randomized studies and 3433 children, found no significant overall mortality reduction with corticosteroids [52]. It reported a reduction in hearing loss overall (RR 0.62, 95% CI 0.47–0.81) and suggested that effects on neurological sequelae differed by corticosteroid dose, with benefit in the 0.6 mg/kg/day subgroup but not in the 0.8 mg/kg/day subgroup. However, it did not provide a robust pneumococcus-specific estimate capable of confirming a mortality or hearing benefit in children. Therefore, the evidence supports a possible benefit in pneumococcal meningitis but does not demonstrate a pathogen-specific pediatric mortality advantage. The severity of pneumococcal inflammation and the suggestive all-age evidence provide a rationale for early empirical dexamethasone, while the magnitude of benefit in children remains uncertain.

Evidence supporting routine adjunctive dexamethasone in meningococcal meningitis is limited and inconsistent. Available analyses have not demonstrated a clear meningococcus-specific benefit for mortality, hearing loss, or other neurological sequelae. Current guidance supports empirical dexamethasone when bacterial meningitis is strongly suspected but recommends reassessment after organism identification; continuation is supported most clearly for pneumococcal or Hib meningitis, rather than confirmed meningococcal disease. Accordingly, dexamethasone should not be routinely continued solely because *N. meningitidis* has been identified. Individualized use may nevertheless be considered with specialist input when the causative organism remains uncertain or when a separate indication for corticosteroid treatment is present [36,45].

Evidence in *L. monocytogenes* meningitis is particularly uncertain. Pediatric comparative data are lacking, and the available adult observational studies have produced conflicting results. Although earlier evidence raised concern about unfavorable outcomes, a prospective nationwide adult cohort subsequently found an association between standard adjunctive dexamethasone and improved outcome [36,45]. Because these data are non-randomized and derived from adults, they do not establish efficacy or safety in children. Dexamethasone should therefore not be described as categorically contraindicated in pediatric *Listeria* meningitis. Rather, routine pathogen-directed use is not supported, and continuation should be determined through specialist assessment, considering the patient’s age, clinical condition, timing of treatment, and any separate corticosteroid indication.

Once microbiological findings become available, the decision to continue treatment should be reassessed. In accordance with current guidance, continuation is most strongly supported when Hib or *S. pneumoniae* is identified. When another organism is confirmed, the potential benefits and risks should be reconsidered in relation to the available pathogen-specific evidence [36,39,45].

### 4.4. Influence of Healthcare and Epidemiological Context

The effectiveness of adjunctive corticosteroid therapy is influenced not only by pathogen biology but also by the epidemiological and healthcare context in which treatment is delivered. In high-income settings, corticosteroids have been associated with reductions in selected neurological complications, particularly sensorineural hearing loss [45,46].

By contrast, randomized trials conducted in low- and middle-income countries have generally failed to demonstrate clear reductions in mortality or broader neurological sequelae [38,45,47]. Potential explanations include delayed presentation, advanced disease at admission, differences in pathogen distribution, malnutrition, HIV co-infection, limited access to intensive care, and reduced availability of timely diagnostic and supportive interventions. Delayed initiation of both antibiotics and corticosteroids may be particularly important because the potential benefit of dexamethasone depends strongly on administration before or with the first antimicrobial dose.

Vaccination has therefore changed both the absolute burden and the etiological composition of pediatric bacterial meningitis. A 99% decline in invasive Hib disease in US children younger than 5 years means that substantially fewer children now present with the pathogen for which the historical corticosteroid-associated hearing benefit was strongest [18,19]. At the same time, pneumococcus remains the most frequently detected of the three major vaccine-preventable pathogens in international pediatric surveillance [20]. Because the pediatric evidence for dexamethasone is less certain in pneumococcal than in Hib meningitis [45,52], the average treatment effect observed in historical Hib-dominated trials cannot be assumed to apply unchanged to contemporary populations. Nevertheless, incomplete vaccine coverage and regional differences in pathogen distribution prevent a single post-vaccination estimate from being generalized worldwide.

This epidemiological transition is not uniform across settings. Hib remains relevant among incompletely vaccinated children, children with impaired vaccine responses, and populations with suboptimal vaccine access or coverage. In highly vaccinated populations, pneumococcal disease, meningococcal disease, neonatal pathogens, and infections in immunocompromised children constitute a larger proportion of cases, but the evidence supporting corticosteroids in these groups is less robust. Pneumococcal serotype replacement, local meningococcal outbreaks, antimicrobial resistance, and differences in access to early diagnosis and intensive supportive care may further modify treatment effect. Consequently, historical relative-effect estimates should not be applied uncritically to current populations; contemporary decisions should consider local pathogen prevalence, vaccination coverage, patient-level vaccination status, age, immune status, and the likelihood that dexamethasone can be administered before or with the first antibiotic dose.

### 4.5. Corticosteroids in Non-Bacterial Meningitis

Evidence concerning corticosteroids in non-bacterial meningitis cannot be extrapolated from acute bacterial meningitis because the responsible pathogens, time of infection, host immune state, mechanisms of tissue injury, and importance of inflammation for pathogen clearance differ substantially. The strength of evidence also varies: randomized evidence supports a short-term survival benefit in tuberculous meningitis and demonstrates harm from routine dexamethasone in HIV-associated cryptococcal meningitis, whereas evidence in viral and other fungal meningitides is sparse and largely observational.

In tuberculous meningitis, adjunctive corticosteroids are supported by randomized evidence, but the certainty differs by outcome. A Cochrane review of nine trials involving 1337 participants found that corticosteroids reduced mortality at 3–18 months by approximately one quarter (RR 0.75, 95% CI 0.65–0.87), an effect rated as high-quality evidence [48]. In contrast, corticosteroids had little or no demonstrated effect on disabling neurological deficit among survivors (RR 0.92, 95% CI 0.71–1.20); this evidence was rated low quality because disability events were less frequent, estimates were imprecise, and outcome definitions and follow-up varied.

Important limitations affect applicability. Only one trial was considered methodologically high quality, while the remaining studies had incomplete reporting or other uncertainties. The evidence was not exclusively pediatric, few participants were HIV-positive, and regimens differed across trials. Moreover, in the single trial reporting five-year outcomes, the earlier mortality advantage was no longer statistically evident (RR 0.93, 95% CI 0.78–1.12), although loss of the apparent effect over time remains unexplained. Corticosteroids can therefore be described as providing a well-supported short- to medium-term survival benefit, while their effects on long-term survival and neurological disability remain less certain [48].

Routine corticosteroid treatment is not recommended for uncomplicated viral meningitis because a clinically meaningful benefit has not been established [11,53]. Corticosteroids may nevertheless be required for a separate indication or for selected immune-mediated complications, but such use should not be considered routine treatment of viral meningitis itself.

Evidence supporting corticosteroids in fungal meningitis is also limited and depends on the causative organism and clinical context. Routine dexamethasone is harmful in HIV-associated cryptococcal meningitis. In the CryptoDex randomized trial, adjunctive dexamethasone did not improve survival and was associated with greater disability, more adverse events, and slower clearance of Cryptococcus from the CSF, leading to early termination of the trial [54]. These findings illustrate why inflammation cannot be assumed to have the same role across meningitis etiologies. In acute bacterial meningitis, rapid pathogen lysis can generate an excessive inflammatory burst that contributes directly to secondary neurological injury. By contrast, cryptococcal meningitis usually develops subacutely in patients with impaired cell-mediated immunity and a high fungal burden. Th1-type cytokines, including interferon-γ and TNF-α, support macrophage activation and fungal clearance; broad immunosuppression may therefore weaken a host response that is already inadequate. In a mechanistic analysis of CryptoDex participants, dexamethasone accelerated the decline in CSF TNF-α, and a faster TNF-α decline correlated with slower fungal clearance. Higher baseline interferon-γ concentrations were also associated with more favorable outcomes. Thus, suppressing inflammation may delay microbiological control without resolving the principal drivers of disease, including fungal burden and elevated intracranial pressure [54]. Corticosteroids should therefore not be used routinely during induction therapy for HIV-associated cryptococcal meningitis. Their use should be reserved for a distinct specialist-defined indication, such as selected inflammatory complications after effective antifungal therapy has been established, rather than for cryptococcal meningitis itself.

The evidence and recommendations for corticosteroid use across bacterial, tuberculous, viral, and fungal meningitis are summarized in Table 4.

### 4.6. Clinical Reassessment and Individualized Treatment

Adjunctive corticosteroid therapy should be regarded as an early, targeted intervention rather than a universal component of treatment for every patient with meningitis. The decision to initiate therapy is commonly made before the causative pathogen is known and should integrate the patient’s age, clinical presentation, suspected etiology, disease severity, timing of antibiotic administration, contraindications, and local epidemiology.

After microbiological identification, treatment should be reassessed according to the causative organism and the strength of the supporting evidence. Continuation is most clearly justified in Hib meningitis and may be appropriate in pneumococcal meningitis, whereas routine continuation is not supported for most other bacterial etiologies. Corticosteroid treatment should also be reconsidered if an alternative diagnosis is established or if adverse effects outweigh the anticipated neurological benefit.

Overall, dexamethasone remains the cornerstone of adjunctive anti-inflammatory therapy for suspected acute bacterial meningitis in children. Its clinical value depends on administration before or with the first antibiotic dose, appropriate pathogen selection, and timely reassessment. Corticosteroids should therefore be used within an individualized, pathogen-informed treatment strategy focused primarily on reducing inflammation-mediated neurological injury rather than improving survival uniformly across all meningitis etiologies.

## 5. Clinical Outcomes of Adjunctive Corticosteroid Therapy in Pediatric Bacterial Meningitis

### 5.1. Mortality and Acute Clinical Outcomes

The effect of adjunctive corticosteroid therapy on mortality and other short-term outcomes in pediatric bacterial meningitis remains limited and inconsistent. Although corticosteroids effectively attenuate the inflammatory response within the central nervous system, current evidence does not demonstrate a reproducible improvement in survival, length of hospitalization, intensive care utilization, or other acute clinical outcomes.

Observational studies have generally failed to identify an independent mortality benefit after adjustment for disease severity and other confounding factors. In a multicenter cohort from the United States, adjunctive corticosteroid therapy was not associated with lower in-hospital mortality or shorter hospitalization; the overall case-fatality rate was 4.2%, and median hospital stay was 12 days in corticosteroid-treated children compared with 10 days in those who did not receive corticosteroids [56]. Similarly, a nationwide Taiwanese cohort reported higher crude mortality, longer hospital stays, and greater hospitalization costs among children treated with corticosteroids. However, these associations were substantially attenuated after multivariable adjustment, suggesting that the unfavorable crude outcomes primarily reflected confounding by indication, with corticosteroids being more frequently administered to children presenting with more severe disease, rather than a direct harmful effect of treatment [57].

More recently, a nationwide Japanese study evaluating early corticosteroid use between 2014 and 2022 likewise found no significant reduction in mortality, intensive care requirements, or duration of hospitalization after adjustment for baseline clinical characteristics [52]. These findings reinforce the observation that any apparent differences in acute outcomes in non-randomized studies must be interpreted cautiously because treatment allocation is strongly influenced by disease severity and clinical presentation.

The absence of a clear survival benefit is also consistent with evidence from randomized controlled trials, systematic reviews, and meta-analyses. Collectively, these studies have not demonstrated a significant reduction in mortality among children receiving adjunctive dexamethasone compared with standard antimicrobial therapy alone [39,43,45,47,52].

Taken together, current evidence indicates that adjunctive corticosteroids should not be administered with the expectation of improving survival or reducing acute healthcare utilization in pediatric bacterial meningitis. Their principal clinical value appears instead to lie in the prevention of selected inflammation-mediated neurological complications, particularly sensorineural hearing loss in specific patient subgroups, as discussed below (Table 5).

### 5.2. Neurological Outcomes

Although adjunctive corticosteroids do not appear to improve survival in pediatric bacterial meningitis, their principal therapeutic rationale is to limit inflammation-mediated neurological injury. Antibiotic-induced bacterial lysis releases cell wall components and other pro-inflammatory mediators into the subarachnoid space, amplifying a cascade characterized by cytokine production, blood–brain barrier disruption, cerebral edema, increased intracranial pressure, cerebral vasculitis, ischemic injury, and neuronal apoptosis [1,2,3,45]. Accordingly, dexamethasone has been investigated primarily as a neuroprotective adjunct rather than as an intervention expected to reduce mortality.

The most consistent neurological benefit of adjunctive corticosteroid therapy is the prevention of sensorineural hearing loss. The Cochrane systematic review, which included 25 randomized controlled trials involving adults and children, demonstrated a significant reduction in hearing impairment among children with Hib meningitis treated with adjunctive dexamethasone, without a corresponding reduction in mortality [43,45]. This finding supports hearing preservation as the best-established clinical benefit of corticosteroid therapy in pediatric bacterial meningitis.

Evidence regarding broader neurological outcomes is less consistent. The meta-analysis by Tian et al. confirmed the absence of a significant survival benefit and reported modest and heterogeneous improvements in neurological sequelae [52]. Importantly, much of the observed benefit originated from studies conducted before the widespread introduction of Hib conjugate vaccination, when Hib accounted for a larger proportion of pediatric bacterial meningitis cases.

Although several randomized controlled trials have suggested modest reductions in overall neurological sequelae with dexamethasone, these findings have not been consistently reproduced in more recent observational cohorts, in which Streptococcus pneumoniae has become a more prominent causative pathogen [52,58]. The changing etiological profile of pediatric bacterial meningitis therefore appears to have reduced the overall magnitude of benefit observed in contemporary clinical practice.

Overall, the neuroprotective effect of adjunctive corticosteroids is most clearly established for hearing preservation, particularly in Hib meningitis. Their effect on other neurological outcomes remains less certain and appears to vary according to pathogen, timing of therapy, and study population.

### 5.3. Long-Term Functional Outcomes

As mortality from pediatric bacterial meningitis has declined with advances in antimicrobial therapy, vaccination, and intensive care, long-term neurological and functional sequelae have become increasingly important determinants of disease burden. Approximately one in five survivors develops at least one permanent neurological disability, emphasizing the substantial contribution of chronic morbidity to the overall impact of the disease [2,15,22,23,24,25,59].

Long-term sequelae encompass a broad range of neurological and neurodevelopmental impairments, including epilepsy, motor deficits, cerebral palsy, cognitive dysfunction, language delay, attention and behavioral disorders, and reduced academic achievement [2,8,9,11,15,22,23,24,25,26]. These complications may persist long after resolution of the acute infection and can substantially affect educational attainment, social functioning, independence, and quality of life.

Several clinical characteristics have been associated with poorer long-term outcomes, including pneumococcal etiology, delayed initiation of antimicrobial therapy, seizures at presentation, altered mental status, increased intracranial pressure, and younger age [38,54]. These factors likely reflect both the severity of the initial inflammatory insult and the extent of secondary neurological injury.

Despite its ability to attenuate acute meningeal inflammation, adjunctive dexamethasone has not been shown to consistently improve broader long-term neurodevelopmental outcomes. Randomized trials have demonstrated a reduction in hearing loss, particularly in children with Hib meningitis, but convincing evidence of benefit for cognitive performance, educational achievement, or overall quality of life remains lacking [52,60].

Interpretation of the available literature is limited by substantial heterogeneity in outcome definitions, differences in the timing and duration of follow-up, and the epidemiological changes associated with widespread Hib vaccination. Many historical trials were conducted in populations and pathogen distributions that differ substantially from those encountered in contemporary practice. Consequently, the long-term neuroprotective effect of adjunctive corticosteroids beyond hearing preservation remains incompletely defined.

Further prospective studies using standardized neurological and neurodevelopmental assessments and sufficiently long follow-up periods are therefore needed to determine whether selected subgroups derive meaningful long-term functional benefit from corticosteroid therapy.

Regardless of adjunctive treatment, children recovering from bacterial meningitis require structured follow-up. International recommendations support post-discharge audiological assessment together with continued neurological and neurodevelopmental surveillance. When deficits are identified, early multidisciplinary rehabilitation, including speech and language therapy, physiotherapy, psychological support, and educational interventions, remains an essential component of comprehensive post-meningitis care [39,45].

### 5.4. Factors Influencing Treatment Effect

The clinical effect of adjunctive corticosteroid therapy is not uniform and is strongly influenced by the timing of administration, causative pathogen, and epidemiological context. Among these factors, treatment timing appears particularly important. Antibiotic-induced bacterial lysis rapidly releases inflammatory bacterial components into the cerebrospinal fluid and amplifies the host inflammatory response. Because a substantial component of secondary neurological injury occurs during this early phase, dexamethasone is most effective when administered immediately before or concomitantly with the first dose of antibiotics [1,3,39,45]. Administration after antimicrobial therapy has already been initiated is associated with diminished or absent benefit.

The magnitude of benefit also varies substantially according to the causative organism. The most consistent signal in the historical pediatric evidence is a reduction in sensorineural hearing loss among children with Hib meningitis, although its magnitude and contemporary generalizability are limited by the age, quality, and heterogeneity of the contributing trials [39,45,47]. However, widespread implementation of Hib conjugate vaccination has profoundly altered the epidemiology of childhood bacterial meningitis. In many high-income countries, *Streptococcus pneumoniae* now accounts for a greater proportion of cases, reducing the number of children presenting with the pathogen for which corticosteroid-associated benefit is most clearly established.

In pneumococcal meningitis, the effect of dexamethasone is less consistent. Some studies suggest a reduction in selected neurological complications, but a clear mortality benefit has not been demonstrated in children [47,57,60]. The therapeutic effect may also be reduced in patients presenting late in the disease course. In addition, less favorable outcomes have been reported in some children with cephalosporin-resistant *S. pneumoniae*, further emphasizing the need for individualized interpretation of potential benefit [61].

Evidence supporting routine adjunctive corticosteroid therapy in meningococcal meningitis remains limited, while no clear benefit has been established in *Listeria monocytogenes* infection. Accordingly, continuation of corticosteroid treatment should be reassessed once microbiological results become available, with particular attention to the identified pathogen and the evidence supporting benefit in that setting [39,45].

Healthcare context may further modify treatment effect. Differences in time to presentation, nutritional status, comorbidities, pathogen distribution, antimicrobial resistance, and access to intensive supportive care may partly explain the smaller or absent benefits reported in some resource-limited settings.

Taken together, these findings indicate that the effectiveness of adjunctive dexamethasone is context-dependent rather than universal. The greatest benefit is observed when treatment is administered before or with the first antibiotic dose and when meningitis is caused by Hib. The transition toward a post-vaccination epidemiological landscape dominated by other pathogens, particularly *S. pneumoniae*, likely explains why contemporary observational studies demonstrate smaller treatment effects than historical randomized trials.

### 5.5. Safety and Clinical Implications

Adjunctive dexamethasone is generally well tolerated when administered as a short course to immunocompetent children with suspected bacterial meningitis. Nevertheless, the available evidence supports relative short-term safety rather than demonstrating an absence of risk. In the 2015 Cochrane review, corticosteroids increased recurrent fever (RR 1.27, 95% CI 1.09–1.47), although no statistically significant increase in other serious adverse events was identified [45]. Similarly, the pediatric meta-analysis by Tian et al. found an increased risk of secondary fever (RR 1.29, 95% CI 1.10–1.51) [52]. Gastrointestinal bleeding, hyperglycemia, electrolyte disturbances, hypertension, and secondary infections were reported infrequently, but individual trials were generally not powered to detect rare harms, and adverse-event definitions and reporting varied across studies [39,43,45,47,52].

The benefit–risk balance may differ substantially among specific pediatric populations. Evidence in neonates is limited to a small number of heterogeneous studies, and differences in causative pathogens, immune responses, pharmacokinetics, and brain development prevent reliable extrapolation from older children. Routine dexamethasone is therefore not supported in neonatal bacterial meningitis. Evidence is also limited for infants aged 28 days to 3 months; in this age group, treatment should be considered only after specialist assessment of the suspected pathogen, clinical severity, and potential risks.

Critically ill children require particular caution because corticosteroid-associated metabolic, gastrointestinal, and infectious complications may be more consequential in the presence of multiorgan dysfunction. Children with shock, coagulopathy, thrombocytopenia, gastrointestinal mucosal injury, poorly controlled hyperglycemia or diabetes, severe malnutrition, or concomitant exposure to ulcerogenic or immunosuppressive treatments require closer clinical and laboratory surveillance. Corticosteroid treatment for catecholamine-resistant septic shock or suspected adrenal insufficiency represents a separate indication and should not be conflated with adjunctive dexamethasone for meningeal inflammation; the corticosteroid selected, dose, and duration may differ.

The safety profile is also less certain in immunocompromised children, including those with HIV infection, malignancy, transplantation, primary immunodeficiency, or prolonged immunosuppressive therapy. These populations were underrepresented in pediatric bacterial meningitis trials and may be at greater risk of secondary infection or impaired pathogen clearance. Particular caution is required when tuberculosis, fungal meningitis, or another opportunistic infection is possible. In HIV-associated cryptococcal meningitis, for example, dexamethasone increased adverse events and disability and slowed CSF fungal clearance [54]. By contrast, tuberculous meningitis is treated with a longer corticosteroid course based on a distinct evidence base and pathophysiological rationale [48,49,51].

Dexamethasone-induced attenuation of meningeal inflammation may also reduce the CSF penetration of certain antimicrobial agents. This concern is especially relevant when resistant pneumococcal meningitis requires vancomycin, although the clinical importance of this interaction remains uncertain. When resistant *S. pneumoniae* is suspected or confirmed, antimicrobial dosing, susceptibility results, microbiological response, and the potential need for specialist-directed treatment adjustments should be carefully assessed [43,61]. Evaluation of corticosteroid suitability must not delay the administration of effective antibiotics.

Safety monitoring should therefore be individualized according to age, immune status, disease severity, comorbidities, concurrent therapies, causative organism, and anticipated corticosteroid duration. Monitoring should include blood glucose, blood pressure, electrolyte concentrations, gastrointestinal bleeding, recurrent fever, new or worsening infection, and neurological status. Once microbiological results become available, dexamethasone should be reassessed and discontinued when pathogen-specific evidence does not support continuation or when patient-specific risks outweigh the anticipated neurological benefit. The principal efficacy, safety, and monitoring considerations for adjunctive dexamethasone are summarized in Table 6.

## 6. Clinical Interpretation and Contemporary Perspectives

Adjunctive corticosteroid therapy in pediatric bacterial meningitis is supported by a strong pathophysiological rationale, but its clinical benefits are selective rather than uniform. Bacterial invasion of the subarachnoid space, followed by antibiotic-induced bacterial lysis, amplifies a host inflammatory response characterized by cytokine release, blood–brain barrier disruption, cerebral edema, intracranial hypertension, vascular injury, and neuronal damage. This inflammatory component provides the biological basis for early anti-inflammatory intervention. Among available corticosteroids, dexamethasone remains the most extensively studied and widely recommended because of its potent anti-inflammatory activity and favorable penetration into inflamed meningeal tissues.

Despite this biological rationale, the clinical benefits of corticosteroids are more limited than might be expected from their anti-inflammatory effects. The most consistent and reproducible benefit is a reduction in sensorineural hearing loss, particularly in children with Hib meningitis when dexamethasone is administered before or concomitantly with the first dose of antibiotics. By contrast, evidence for benefit in pneumococcal meningitis is less consistent, while data supporting routine use in meningococcal disease remain limited. Importantly, available studies have not demonstrated a clear survival advantage in children.

Timing of administration is a major determinant of treatment efficacy. The inflammatory response is rapidly intensified following antibiotic-induced bacterial lysis, and the potential neuroprotective effect of dexamethasone therefore appears greatest when treatment is initiated immediately before or at the same time as antimicrobial therapy. Delayed administration, once the inflammatory cascade is already established, is associated with reduced or absent benefit. This timing dependency reinforces the concept that corticosteroids act primarily by attenuating the early inflammatory burst rather than by modifying the underlying infection itself.

The contemporary relevance of corticosteroid therapy must also be interpreted in light of major changes in the epidemiology of pediatric bacterial meningitis. Much of the evidence supporting dexamethasone originated from studies conducted when Hib was a leading cause of childhood meningitis. Widespread Hib and pneumococcal vaccination has substantially altered pathogen distribution and reduced the proportion of children with infections for which corticosteroid-associated benefit is most clearly established. Consequently, the magnitude of benefit observed in contemporary cohorts is generally smaller than that reported in historical randomized trials.

Geographical and healthcare-related factors further contribute to variability in treatment effect. Studies conducted in low- and middle-income countries have not consistently demonstrated reductions in mortality or neurological sequelae. Differences in timing of presentation, disease severity, pathogen distribution, nutritional status, comorbidities, and access to intensive supportive care may partly explain these discrepancies. These observations suggest that the efficacy of corticosteroids depends not only on the biological characteristics of the infection but also on the clinical and healthcare context in which treatment is delivered.

Another important consideration is the distinction between preventing specific neurological complications and improving overall disease outcomes. Adjunctive corticosteroids have not been consistently associated with reductions in intensive care requirements, duration of hospitalization, or overall disease severity. Moreover, although dexamethasone may reduce hearing loss in selected patients, evidence supporting meaningful improvements in broader long-term neurodevelopmental outcomes, including cognition, functional recovery, and quality of life, remains limited. Corticosteroids should therefore be regarded primarily as a targeted neuroprotective adjunct rather than as a disease-modifying or life-saving therapy.

Taken together, the available evidence supports a selective and context-dependent approach to adjunctive corticosteroid therapy in pediatric bacterial meningitis. Treatment decisions should integrate the suspected or confirmed causative pathogen, timing of presentation and antibiotic administration, disease severity, vaccination-era epidemiology, and local healthcare context. The clearest benefit remains the prevention of sensorineural hearing loss in appropriately selected children, particularly those with Hib meningitis treated early. In contrast, routine corticosteroid administration across all etiologies cannot be justified by the available evidence. Future studies should therefore focus on identifying the pediatric subgroups most likely to benefit, defining biomarkers of excessive inflammatory activity, and determining whether targeted anti-inflammatory strategies can improve long-term neurological and neurodevelopmental outcomes in the contemporary post-vaccination era.

Future adjunctive treatment is likely to move from uniform suppression of inflammation toward pathogen-informed and host-response–guided therapy. Rapid molecular pathogen identification, combined with measurements of systemic and CSF inflammatory activity, could help distinguish children likely to benefit from early immunomodulation from those in whom inflammation is less prominent or corticosteroid-associated risks predominate. Candidate approaches include cytokine and chemokine panels, markers of blood–brain barrier injury, neuronal and glial injury biomarkers, and proteomic or metabolomic signatures associated with neurological complications. Recent pediatric CSF proteomic studies have identified multivariable protein signatures capable of distinguishing bacterial meningitis with neurological complications from uncomplicated disease, but these findings require prospective external validation before they can guide treatment.

Emerging therapeutic strategies also include more selective modulation of inflammatory pathways, including complement activation, leukocyte recruitment, inflammasome signaling, oxidative injury, and endothelial or blood–brain barrier dysfunction. Additional experimental approaches seek to neutralize pathogen-specific toxins, improve intracranial delivery of neuroprotective agents, or protect particularly vulnerable structures such as the cochlea and hippocampus. Such interventions may theoretically attenuate neurological injury while preserving immune mechanisms required for pathogen clearance. At present, however, most remain preclinical or supported only by early translational evidence.

Clinical development should therefore prioritize multicenter, pathogen-stratified and risk-enriched trials rather than treating pediatric bacterial meningitis as a single biological entity. Adaptive designs may be particularly useful given the declining incidence of individual vaccine-preventable etiologies. Trials should incorporate standardized hearing, cognitive, behavioral, functional, and quality-of-life outcomes with follow-up extending beyond hospital discharge. Parallel evaluation of treatment accessibility in low-resource settings will also be necessary to ensure that emerging precision strategies do not widen existing disparities in meningitis outcomes.

## 7. Conclusions

Adjunctive dexamethasone should not be used uniformly across all pediatric meningitis etiologies. In children older than 3 months with strongly suspected bacterial meningitis, dexamethasone may be initiated at 0.15 mg/kg intravenously every 6 h, up to 10 mg per dose, for 4 days. The first dose should be administered immediately before or concomitantly with the first antibiotic dose; antimicrobial treatment must not be delayed to administer dexamethasone. When corticosteroid initiation occurs after antibiotics, the expected benefit is less certain and specialist advice is appropriate, particularly if more than 12 h have elapsed.

Once the causative organism is identified, continuation is most clearly supported for Hib meningitis, for which reduction in sensorineural hearing loss is the best-established pediatric benefit. Continuation may also be appropriate for pneumococcal meningitis, although pediatric evidence is less consistent and a mortality benefit has not been established. Routine continuation is not supported for confirmed meningococcal meningitis or other bacterial etiologies. Evidence in pediatric *L. monocytogenes* meningitis is insufficient and adult observational findings are conflicting; treatment should therefore be individualized with specialist input rather than described as categorically contraindicated. Evidence is also insufficient to recommend routine corticosteroid treatment in neonates.

Thus, the role of dexamethasone in pediatric bacterial meningitis is best understood as early, pathogen-informed neuroprotection rather than universal therapy. Future studies should determine whether biomarkers of inflammatory and neurological injury can improve patient selection and should evaluate pathogen-specific or targeted immunomodulatory strategies using standardized long-term hearing, neurodevelopmental, functional, and quality-of-life outcomes.

## Figures and Tables

**Figure 1:**
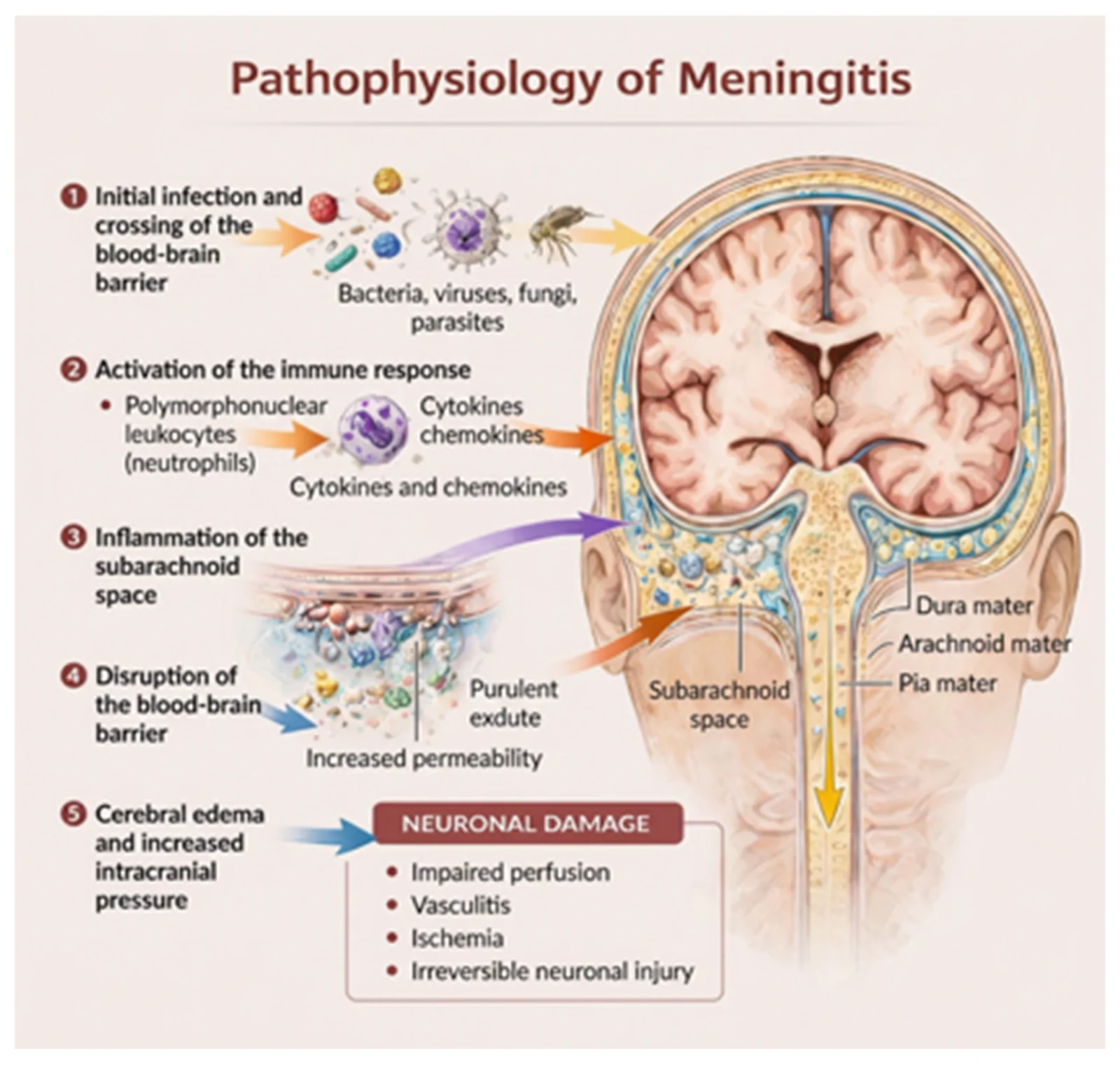
Pathophysiology of bacterial meningitis and mechanisms leading to neurological injury. After hematogenous dissemination, pathogens cross the blood–brain barrier and enter the cerebro-spinal fluid (CSF), where bacterial replication and antibiotic-induced lysis release patho-gen-associated molecular patterns. Activation of resident immune cells induces the production of pro-inflammatory cytokines and chemokines, including interleukin (IL)-1beta, tumor necrosis fac-tor-alpha (TNF-alpha), CXCL1, CXCL2, and CXCL5, which promote neutrophil recruitment and the release of reactive oxygen species and proteolytic enzymes. The resulting increase in blood–brain barrier permeability, purulent exudate, cerebral edema, raised intracranial pressure, vasculitis, im-paired perfusion, and ischemia contributes to neuronal and cochlear injury. Adjunctive dexame-thasone acts principally at the cytokine and chemokine amplification stage depicted in step 2 by suppressing inflammatory gene transcription and leukocyte trafficking; when used, it should be administered immediately before or with the first antibiotic dose to attenuate downstream second-ary injury. Corticosteroids are adjunctive and do not eradicate the infecting pathogen. The figure has been created by the authors; no AI-based image-generation software was used.

**Table 1:** Major etiological agents of meningitis according to age group.

Age Group	Most Common Pathogens	References
Preterm neonates	*Escherichia coli*, Group B *Streptococcus*	[27,28]
Neonates and young infants (<3 months)	Group B *Streptococcus*, *Escherichia coli*, *Listeria monocytogenes*, *Streptococcus pneumoniae*	[27,28,29,30]
Infants and children (3 months–10 years)	*Streptococcus pneumoniae*, *Neisseria meningitidis*, *Haemophilus influenzae* type b (in settings with incomplete vaccination coverage)	[2,23,31,32]
Adolescents	*Neisseria meningitidis*, *Streptococcus pneumoniae*	[23,31,32]
Adults	*Streptococcus pneumoniae*, *Neisseria meningitidis*	[1,31,32]
Older adults and immunocompromised patients	*Streptococcus pneumoniae*, *Listeria monocytogenes*, Gram-negative bacilli	[1,33]

**Table 2:** Integrated algorithm for the initial management of suspected bacterial meningitis in children.

Stage	Recommended Actions	Key Considerations
Recognition and triage	Suspect meningitis in a child with compatible systemic or neurological features; arrange immediate admission or urgent transfer to a facility able to perform lumbar puncture, provide parenteral therapy, and manage severe illness.	Presentations may be nonspecific in neonates and infants. Do not wait for the complete classic triad before initiating assessment and treatment [1,36,39].
Stabilization	Assess airway, breathing, circulation, neurological status, capillary glucose, and signs of sepsis. Obtain intravenous or intraosseous access and treat shock, hypoglycemia, hypoxemia, and seizures promptly.	Seek early pediatric intensive care support for cardiorespiratory instability, recurrent seizures, declining consciousness, or suspected intracranial hypertension [1,12,36,39].
Infection control	Initiate droplet precautions when meningococcal disease is suspected and notify public health authorities as required.	Arrange antimicrobial prophylaxis for eligible close contacts according to local public health guidance [12,36].
Blood investigations	Obtain blood cultures, paired blood glucose, complete blood count, inflammatory markers, electrolytes, and renal and hepatic function tests. Add coagulation studies when clinically indicated.	Collect samples before antibiotics when feasible, but never delay empirical treatment to complete investigations [12,36,39,40].
Lumbar puncture	Perform promptly when clinically safe. Assess CSF appearance, leukocyte count and differential, red blood cell count, protein, glucose with paired blood glucose, Gram stain, culture and susceptibility, and molecular tests when available.	Defer temporarily in cardiorespiratory instability, marked impairment of consciousness, focal neurological signs, papilledema, uncontrolled bleeding risk, infection at the puncture site, or suspected intracranial mass effect [1,12,36,39].
Neuroimaging	Reserve cranial imaging before lumbar puncture for children with clinical features suggesting a space-occupying lesion, obstructive hydrocephalus, or increased risk of cerebral herniation.	Routine imaging before lumbar puncture is not recommended. When imaging or lumbar-puncture deferral is necessary, obtain blood cultures and begin treatment immediately [12,36,39].
Empirical antibiotics	Start intravenous antimicrobial treatment as early as possible, ideally within 1 h of hospital assessment. Use an age-appropriate third-generation cephalosporin-based regimen, adding vancomycin or ampicillin when indicated.	Select therapy according to age, immune status, clinical context, local resistance patterns, and neonatal protocols. Diagnostic uncertainty, imaging, or transfer must not delay antibiotics [2,12,36,39,41,42,43,44].
Adjunctive dexamethasone	When indicated, administer dexamethasone immediately before or with the first antibiotic dose.	Do not delay antibiotics. Reassess continuation after pathogen identification; the clearest pediatric benefit concerns hearing preservation in Hib meningitis [1,12,36,39].
Supportive treatment	Use isotonic fluids when intravenous fluids are required; treat seizures and shock; and monitor sodium, glucose, perfusion, urine output, consciousness, and signs of intracranial hypertension.	Avoid routine fluid restriction and routine glycerol. Hypertonic saline or mannitol may be used temporarily for suspected acute intracranial hypertension while definitive management proceeds [12,36,39].
Microbiological reassessment	Review culture, molecular, and antimicrobial-susceptibility results and narrow therapy to the most appropriate pathogen-directed regimen.	Individualize treatment duration according to the organism, clinical response, complications, and adequacy of source control [12,36,39].
Sequelae assessment	Arrange neurological and audiological assessment before discharge or during early follow-up, with multidisciplinary referral when abnormalities are identified.	Audiological evaluation should preferably occur before discharge or within 4 weeks. Initiate hearing, neurological, developmental, and rehabilitation services early when required [12,36,39].

Abbreviations: CSF, cerebrospinal fluid; Hib, *Haemophilus influenzae* type b; IV, intravenous.

**Table 3:** Corticosteroids evaluated in meningitis: mechanisms, indications, and clinical considerations.

Corticosteroid	Main Mechanism of Action	Principal Indications	Advantages	Limitations
Dexamethasone [1,3,12,36,39,45]	Suppression of pro-inflammatory cytokines (TNF-α, IL-1β), reduction in leukocyte migration and cerebral edema	Acute bacterial meningitis	Best evidence base; excellent CNS penetration; guideline-recommended	Benefit dependent on early administration
Prednisolone [11,48,49,50,51]	Broad anti-inflammatory and immunomodulatory effects	Tuberculous meningitis	Useful for prolonged treatment courses	Limited evidence in acute bacterial meningitis
Hydrocortisone [12,48]	Anti-inflammatory and glucocorticoid activity	Historical studies; adrenal insufficiency	Widely available	Limited evidence for routine meningitis treatment
Methylprednisolone [12]	Potent anti-inflammatory activity	Selected severe CNS inflammatory conditions	Potential alternative in refractory cases	Insufficient evidence for routine use
Fludrocortisone [12,39]	Mineralocorticoid activity	Septic shock with adrenal insufficiency	Hemodynamic support	No direct role in reducing meningeal inflammation

Abbreviations: CNS, central nervous system.

**Table 4:** Role of adjunctive corticosteroid therapy according to meningitis etiology.

Etiology	Recommended Corticosteroid	Evidence of Benefit	Main Clinical Outcomes	Recommendation
Pneumococcal meningitis [2,39,45]	Dexamethasone	Moderate–Strong	Reduction in neurological complications; uncertain effect on mortality in children	Recommended
*Haemophilus influenzae* type b meningitis [39,45,47]	Dexamethasone	Strong	Significant reduction in sensorineural hearing loss	Strongly recommended
Meningococcal meningitis [39,45]	Dexamethasone	Limited; no consistent pathogen-specific benefit demonstrated	Pediatric evidence does not support routine continuation after microbiological confirmation	Not routinely recommended after confirmation; individualized specialist assessment only when the organism remains uncertain or another indication exists
Tuberculous meningitis [11,48,49,50,51]	Dexamethasone or Prednisolone	Strong	Improved survival; limited impact on long-term neurological disability	Recommended
Viral meningitis [11,53]	Not routinely indicated	Weak/Absent	No proven clinical benefit	Not recommended
Cryptococcal meningitis [54]	Not recommended	Negative	Increased adverse events and poorer outcomes	Contraindicated/Discouraged
*Listeria* meningitis [39,55]	Not recommended	Very limited pediatric evidence; conflicting adult observational findings	No pediatric trial evidence establishes benefit or harm; adult findings should not be extrapolated directly	Not routinely recommended; specialist-guided individualized decision

**Table 5:** Clinical outcomes associated with adjunctive dexamethasone therapy in pediatric bacterial meningitis.

Outcome	Effect of Adjunctive Dexamethasone	Strength of Evidence	Comments
Mortality [39,43,45,47,52,56,57]	No consistent reduction	Moderate	No significant benefit demonstrated in randomized controlled trials or adjusted observational studies.
Sensorineural hearing loss [39,43,45,47]	Significant reduction (mainly in *Haemophilus influenzae* type b meningitis)	High	Greatest benefit when dexamethasone is administered before or with the first antibiotic dose.
Overall neurological sequelae [45,47,52,58]	Modest reduction	Moderate	Benefit mainly observed in historical studies conducted before widespread Hib vaccination.
Length of hospital stay [52]	No consistent reduction	Low	Findings remain inconsistent across observational studies.
ICU admission/Mechanical ventilation [52,56,57]	No consistent reduction	Low	No independent benefit after adjustment for disease severity.
Healthcare costs [52,57]	No consistent reduction	Low	Higher crude costs largely reflect confounding by indication.
Adverse events [39,43,45,47,52]	Generally well tolerated	Moderate	Serious adverse events are uncommon and rarely require discontinuation of therapy.

**Table 6:** Practical clinical implications of adjunctive dexamethasone therapy in pediatric bacterial meningitis.

Clinical Aspect	Current Evidence	Practical Implication	References
Mortality	No consistent reduction	Corticosteroids should not be administered with the expectation of improving survival.	[39,43,45,47,52,56,57]
Neurological sequelae	Modest benefit	Adjunctive dexamethasone may reduce inflammation-mediated neurological injury in selected patients.	[45,47,52,58]
Sensorineural hearing loss	Strongest evidence of benefit, particularly in Hib meningitis	Early administration remains the principal indication for adjunctive therapy.	[39,43,45,47]
Timing of administration	Critical determinant of efficacy	Dexamethasone should be administered immediately before or together with the first dose of antibiotics whenever possible.	[1,3,39,45]
Pathogen-specific effect	Benefit varies according to the causative organism	Greatest evidence supports use in Hib meningitis; benefit is less consistent in pneumococcal disease and limited in meningococcal meningitis; generally not recommended once *Listeria monocytogenes* infection is confirmed.	[39,45,47,52,55,60]
Safety	Generally well-tolerated during short-course treatment, but recurrent or secondary fever is increased and evidence for rare adverse events is limited	Monitor glucose, blood pressure, electrolytes, gastrointestinal bleeding, recurrent fever, and secondary infection. Closer monitoring and individualized assessment are required in neonates and young infants, critically ill or immunocompromised children, and patients with shock, coagulopathy, metabolic disorders, or suspected opportunistic infection	[39,43,45,47,52]
Current role in clinical practice	Selective use	Treatment decisions should be individualized according to the suspected pathogen, timing of antibiotic administration, disease severity, and local epidemiology.	[39,45,47,52,60]

## Data Availability

No new data were created or analyzed in this study. Data sharing is not applicable.
